# On Severe Cold or Congelation as a Remedy of Disease

**Published:** 1849

**Authors:** James Arnott


### ARTICLE III.

*On Severe Cold or Congelation as a Remedy of Disease.* By JAMES ARNOTT, M. D.

Many powerful physical agents which are destructive when they act in an uncontrolled manner on the human body, become remedial when their action is regulated and applied under appropriate circumstances. Excessive heat, which when extensively applied to the body, is immediately destructive of life, may be so limited or controlled, even when it is of so high a degree as to render iron white, as to furnish a useful therapeutical means; and the opposite extreme of temperature, or a degree of cold causing congelation of the animal textures, which has hitherto been only regarded as the cause of disease, when it is not too low, too extensive, or too much prolonged, constitutes, as will appear by the following observations, a remedy of great importance, and of very general application.

Intense cold or congelation would probably, long ere now, have obtained a place amongst the more potent therapeutical means, but for a mistaken notion respecting its effects on the animal structure. It has been always dreaded as a cause either of violent reaction and inflammation, or, if longer continued, of the immediate gangrene or death of the part subjected to it; and the common accidents from intense frosts in severe winters and high latitudes, have appeared to justify this apprehension. But, although it is perfectly true that the body thus exposed to intense heat, or is burned by accidental fires, yet when severe cold is regulated as has been just described, it becomes an agent of a very different character: it produces neither reaction nor mortification. When limited in degree, duration, and extent, it exerts an anti-inflammatory power; it appears to depress the vascular and nervous energies permanently, and yet within the bounds of safety; and, probably, while it depresses, it considerably modifies the vital action.

I was led to doubt the correctness of the common opinion of the effects of congelation, by observing that when intense cold had been used to remove the sensibility of parts previously to surgical operations, the wound appeared, in every instance, to heal more rapidly than under the usual circumstances; and I had no hesitation in endeavoring to extend the useful application of so valuable a remedy.

As it is my only wish at present to establish the right of congelation to be admitted amongst our more valuable remedies, I will not enter into details respecting the diseases in which I have had recourse to it. If the above explanation of its action be correct, it is obviously applicable to a great number of the more formidable maladies to which the human frame is subject. As respects its anæsthetic action on the nerves, it exerts a most beneficial influence in many painful diseases, the seat of which can be reached by it; but it is probably as a preventive and prompt remedy of vascular excitement or inflammation, that it will be chiefly valued. Cold has already a high character as a remedy for inflammation; but a continuous low degree of cold, such as has hitherto beeen employed, (or rather which it has been the endeavor to employ,) may only repress the morbid energy, which a short application of a much greater degree of cold, may altogether and at once destroy. A class of diseases in which both nerves and blood-vessels are in a morbid condition, are affections of the skin, and these were, naturally, from being so obviously under the influence of the new remedy, amongst the first in which in which it was used. The most obstinate cutaneous diseases have yielded to congelation so speedily as almost, with respect at least to some of them, to suggest another explanation of its modus operandi. Had the cases of prurigo so treated been dependent, like scabies, upon the presence of parasitic animals, they could not have been more speedily cured by the sudden extinction of the life of these animals in the cold. A most distressing attack of prurigo pudendi was completely cured by two congelations, each of about thirty seconds duration, after a prussic acid lotion, and other routine applications, had been tried in vain.

Congelation to the degree which has been specified may be produced by the common frigorific mixture of ice and salt; though for particular purposes, one of greater power might be prepared. The easiest mode of using the frigorific is, to dip a piece of ice into salt, and then apply it closely to the part. Congelation will be thus produced in half a minute. When the surface to be frozen is irregular, a little pounded ice and salt may be placed on a rag or on a flat bit of sponge, or the mixture may be confined to the part by a deep ring or a bottomless cup, made of gutta percha or bees-wax, or by means of a thin bladder, each being provided with a small tube carry off the warmer brine as the ice dissolves. The application of ice or very cold water to the skin is painful, but the frigorific mixture immediately suspends the sensibility.

It has been applied to a carious tooth, an inflamed and ulcerated mouth from mercurial ptyalism, and an irritable ulcer, without causing uneasiness; but when the congelation commences, there is, for a few seconds, an uneasy sensation of contraction, proportionate to its degree. In the case of ptyalism referred to, (a patient of the Brighton Dispensary, who had been deprived of sleep for two nights by the affection of the mouth,) there was no return of pain, except in mastication, after one application of the frigorific, which was sufficiently powerful to blanch the lower lip as it flowed over it.

As the prevalent erroneous notion, that congelation of the animal textures, must, in every instance, produce either violent reaction or gangrene, will probably prove some impediment to the reception of this important therapeutic agent, it may be well to remind such as may object to it on this account, of the vast difference between intense cold, acting for a long period on the extreme parts of the body where the circulation of the blood is never vigorous, and ready to reanimate the parts in which it has ceased. There is as great a difference between these cases as would exist between that of opium taken in unlimit ed quantity by a feeble child, and taken in a suitable dose by an adult. I have now employed congelation nearly a hundred times for anæsthetic and remedial purposes, with out its being followed in a single instance by any injurious effect. Even if the congelation be kept up for several minutes there is no worse consequences than slight congestion, with redness of a few days continuance.

As respects the employment of severe cold for the production of local anæsthesia, it may be remarked, that although a fatal result now and then from the use of chloroform may not be thought to be, by the great mass of physicians, a sufficient objection to its use, and although the intoxication or loss of consciousness during its action may be only deemed a slight inconvenience, still the facilitating of the healing process, by the prevention of an injurious degree of inflammation ought, I think, to entitle the application of cold to the preference in the great majority of surgical operations.

